# Extramedullary Relapse of the AML Transformed from MDS Following Auto-HSCT: A Case Report

**DOI:** 10.1007/s12013-014-9926-3

**Published:** 2014-04-03

**Authors:** Jin Wang, Yu Liu, Xu Zhou, Zheng Li, Xiang Li, Hualiang Xiao

**Affiliations:** 1 Department of Hematology, Institute of Surgery Research, Daping Hospital, Third Military Medical University, Chongqing, 400042 China; 2 Department of Pathology, Institute of Surgery Research, Daping Hospital, Third Military Medical University, Chongqing, 400042 China

**Keywords:** Acute myeloid leukemia, Myelodysplastic syndrome, Extra-medullary relapse, Stem cell transplant, Auto-HSCT, AML

## Abstract

The treatment for AML (Acute myeloid/myelogenous leukemia) transformed from MDS (myelodysplastic syndrome) is difficult and controversial clinically, especially in elder patients. In this case report, we diagnosed a 59-year-old female patient with AML-M2a transformed form MDS which might be caused by her chemotherapy for mastocarcinoma. After achieving complete remission (CR) through combined chemotherapy, autologous peripheral blood stem cell transplantation (auto-PBSCT) was attempted. Following auto-HSCT, marrow showed continuous CR but the patient later developed extramedullary bone-infiltration relapse. Then local radiotherapy has been applied, and the patient now has prolonged survival. This is the first (or a successful) case report of auto-HSCT in an elderly patient with AML transformed from MDS.

## Background

Acute myeloid/myelogenous leukemia (AML) is a cancer of the myeloid line of blood cells which is characterized by rapid growth and accumulation of leukocytes in the bone marrow and interference with the production of normal blood cells. AML is the most common type of acute leukemia affecting adults, and its incidence generally increases with age. AML in older adults has its main characteristics as pancytopenia and myeloproliferative hyperplasia to hypoplasia. Some patients have the history of early myelodysplastic syndrome (MDS); most of them have an occult onset and, therefore, do not seek medical help until it evolves into AML [1, 2]. Given that, the disease is difficult to deal with and most patients have a poor prognosis. Stratified therapy and individual therapy are the principles of AML treatment. As regard the intermediate and high risks of normal or simple karyotypic aberration, anti leukemia combined chemotherapy can be adopted [3]. Besides, concerning the relatively younger patients in a better general condition, and provided the HLA-matched suitable sibling donors are available, allogeneic bone marrow transplantation can still be considered [4]. Here, we report a case of the 59-year-old female patient diagnosed with AML-M2a. There was a history of combined chemotherapy of postoperative mastocarcinoma. Before onset, repeat blood examinations indicated cytopenia but the patient did not seek appropriate medical intervention. At the time of present diagnosis, fluorescence in situ hybridization (FISH) test detected MDS related to abnormal chromosomal translocation, and the patient was considered suffering from acute leukemia (AL) transformed from MDS. After achieving complete remission (CR) through combined chemotherapy, autologous peripheral blood stem cell transplantation (auto-PBSCT) was attempted. After surgery, marrow showed continuous CR but the patient was complicated by extramedullary bone-infiltration relapse. The case report is as follows:

## Case History and Clinical Information

### Admission and Diagnosis

**Fig. 1:**
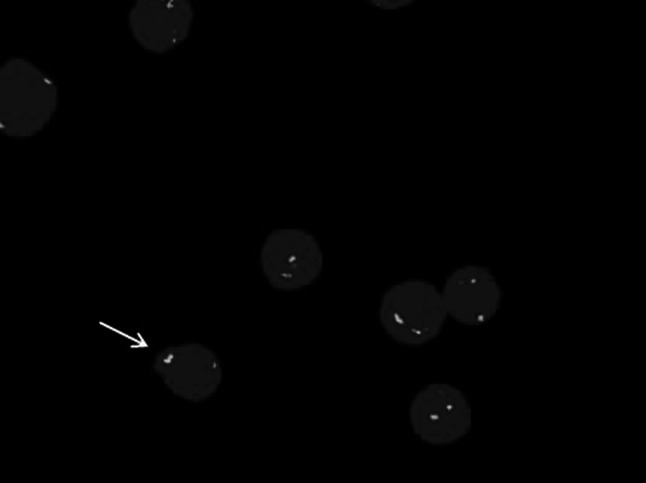
D7S486/CSP7 deletion. The D7S486/CSP7 deletion is shown which was observed in 78.7 % of the abnormal cells

**Fig. 2:**
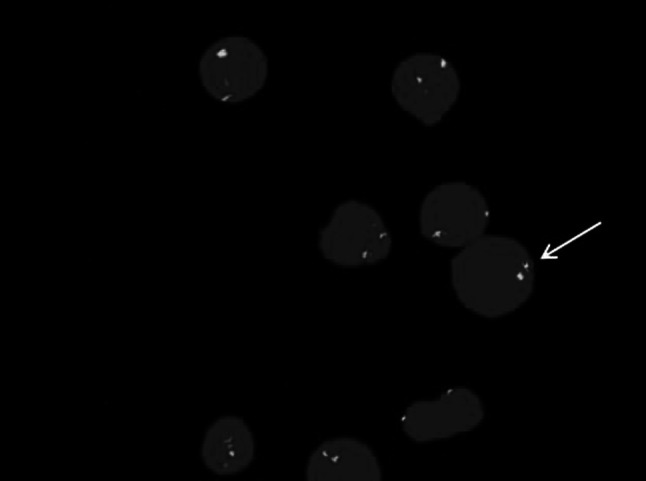
D7S522/CSP7 deletion. The D7S522/CSP7 deletion is shown which was observed in 83.9 % of the abnormal cells

**Fig. 3:**
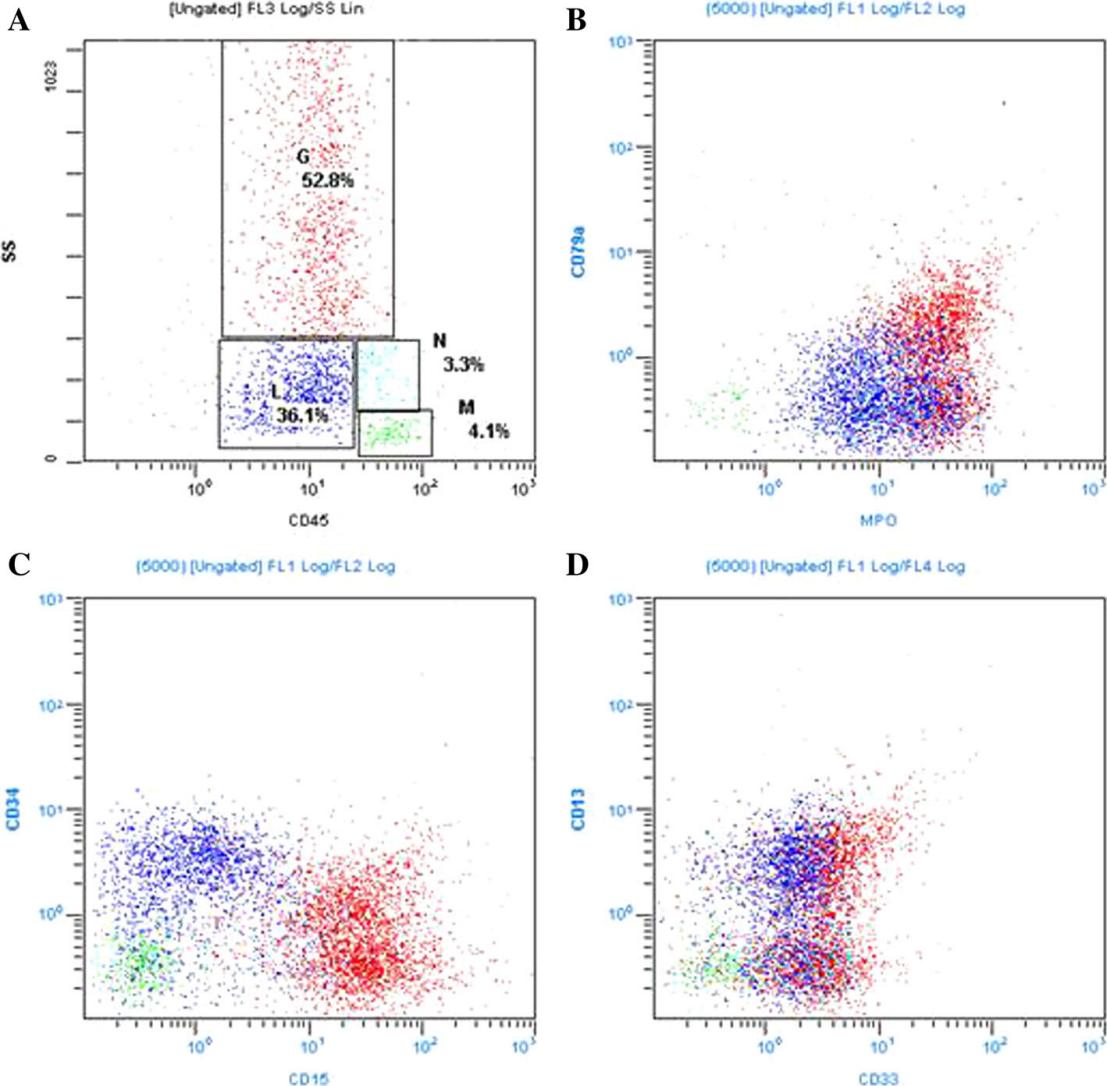
Primordial cell immunophenotyping (**a**–**d**). Primordial phenotypic features were: CD34+CD33+CD13+MPO+HLA-DR+

Female patient, 59 years old, having dizziness and fatigue associated with palpitations after physical activities and other symptoms of anemia for more than one month, was admitted to our hospital on November 23, 2010 while in fever for almost a day. Previously, in 1994, the patient was diagnosed with right breast cancer and had undergone radical resection. After the surgery, she had two chemotherapy courses of cyclophosphamide, methotrexate, and fluorouracil pyrimidine. In the last three years before recent admission, the patient was subjected to routine blood testing which revealed a mild reduction of white blood cells (WBC), red blood cells, and platelets (PLT) but no further examination was conducted. The present blood analysis indicated as follows: WBC: 0.78 × 10^9^/L; Hemoglobin (Hb): 49 g/L; PLT: 40 × 10^9^/L; myeloblasts accounted for 10 %. Myeloproliferative hyperplasia: myeloid accounted for 81.5 %, erythroid accounted for 5 %, of which, myeloblasts accounted for 76 %. FISH showed negative AML1/ETO; D7S486/CSP7 deletion: abnormal cells accounted for 78.7 % (Fig. 1); D7S522/CSP7 deletion: abnormal cells accounted for 83.9 % (Fig. 2). Myelocytes immunophenotyping by flow cytometry: there were increasing initial myelocytes in primordium region, myeloblasts and immature granulocytes in granulocytic region, and the primordial phenotypic features were CD34+CD33+CD13+MPO+HLA-DR+. Conclusion: AML-M2 (Fig. 3a–d).

Flow cytometry (FCM) results showed blue primordial immunophenotyping as CD19+CD33+CD13+HLA-DR++MOP+CD15+. In this group of cells, partial CD13 and CD15 had weak expression; most CD19, CD33, CD13, MPO, and CD34 had weak expression, and a vast majority of HLA-DR had intermediate expression.

### Therapy

From December 2nd to December 16th, we introduced Harringtonine/Ara-C/G-CSF (HAG) regimen in remission induction therapy as follows: H (Harringtonine) 1.5 mg/d, VD, d1-d14; Ara-C (cytosine arabinoside) 10 mg/m^2^, 1H, d1–d14; G-CSF (Granulocyte colony-stimulating factor) 375 μg/d, 1H1 d0–d14. Blood test results at 2 weeks after chemotherapy showed as follows: WBC: 6.03 × 10^9^/L; Hb: 64 g/L; PLT: 333 × 10^9^/L; the marrow review showed a complete remission. The HA (Harringtonine plus cytosine arabinoside) regimen and DA (daunorubicin plus cytosine arabinoside) regimen were introduced, respectively, as consolidation chemotherapy.

Since there was no HLA-matching donor available for this patient, and in view of the risks involved in allogeneic stem cell transplantation (allo-SCT), auto-PBSCT was suggested in order to achieve a greater benefit of strengthening treatment and possible cure. In April 2011, the patient was given strengthening therapy, HSCT mobilization, auto-PBSCT collection, and in vitro cryopreservation. The adopted regimen was reduced mitoxantrone (Mit: 8 mg/d d1-d3) + intermediate dose of cytosine arabinoside (ID-Ara-C: 1.0 g/d, once/12 h, d1; 0.5 g/d, once/12 h, d2-d3) + G-CSF regimen to mobilize peripheral blood stem cells. G-CSF was administered (375 μg/d) when granulocytes fell to a lower level. 1H Fresenius blood cell separator was used to collect peripheral blood cells, and a total of mononuclear cells (MNC) 7.41 × 10^8^/kg body weight, and CD34 + cells 4.04 × 10^6^/kg body weight were collected. Cryopreservation fluid was added to the blood cells and stored in freezer at −80 °C. The cryopreservation fluid comprised of 5 % DMSO, 3 % hydroxyethyl starch, and 2 % albumin.

Before the transplantation, routine blood results were as follows: WBC: 3.48 × 10^9^/L; Hb: 116 g/L; PLT: 149 × 10^9^/L; marrow examination and flow cytometry immunophenotyping were both normal. In August 2011, auto-PBSCT was executed; pretreatment regimen adopted reduced BU plus CY, i.e., BU (busulfan) 0.7 mg/m^2^/6 h, −6d to −3d; CY (cyclophosphamide) 42 mg/kg body weight, −2d to −1d.

**Fig. 4:**
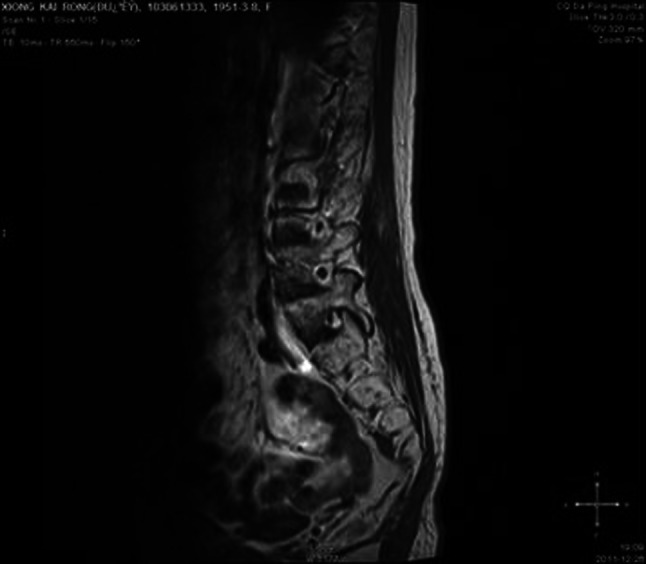
Magnetic resonance imaging (MRI) scan. MRI prompted the vertebral bone destruction

**Fig. 5:**
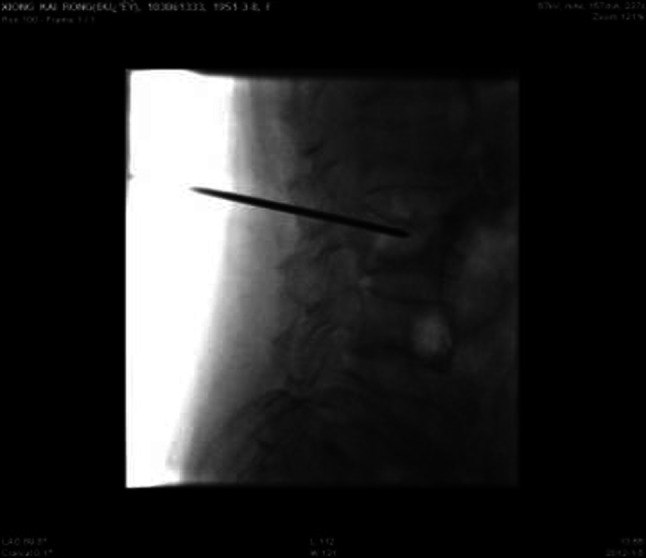
Vertebral puncture biopsy

**Fig. 6:**
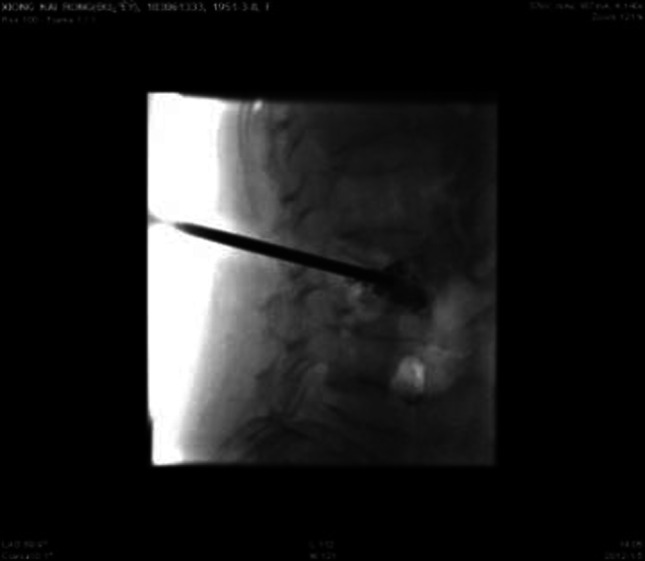
Injection of bone cement

**Fig. 7:**
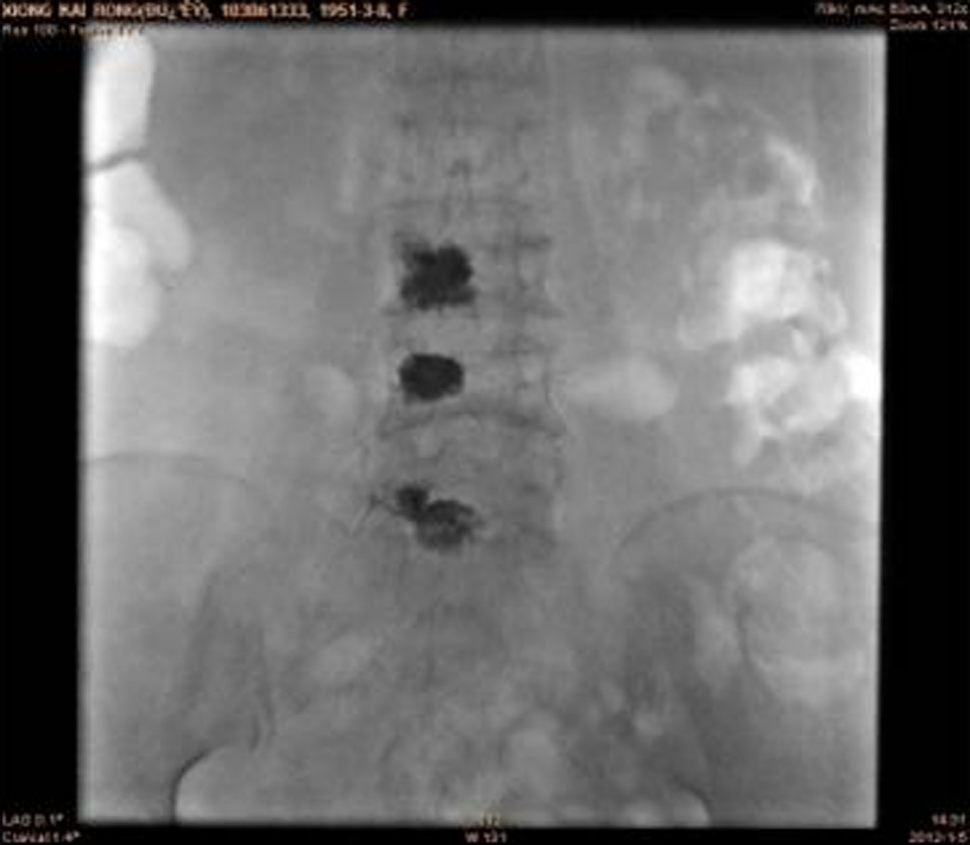
Bone cement injection after VP of 3rd, 4th, and 5th vertebrae

**Fig. 8:**
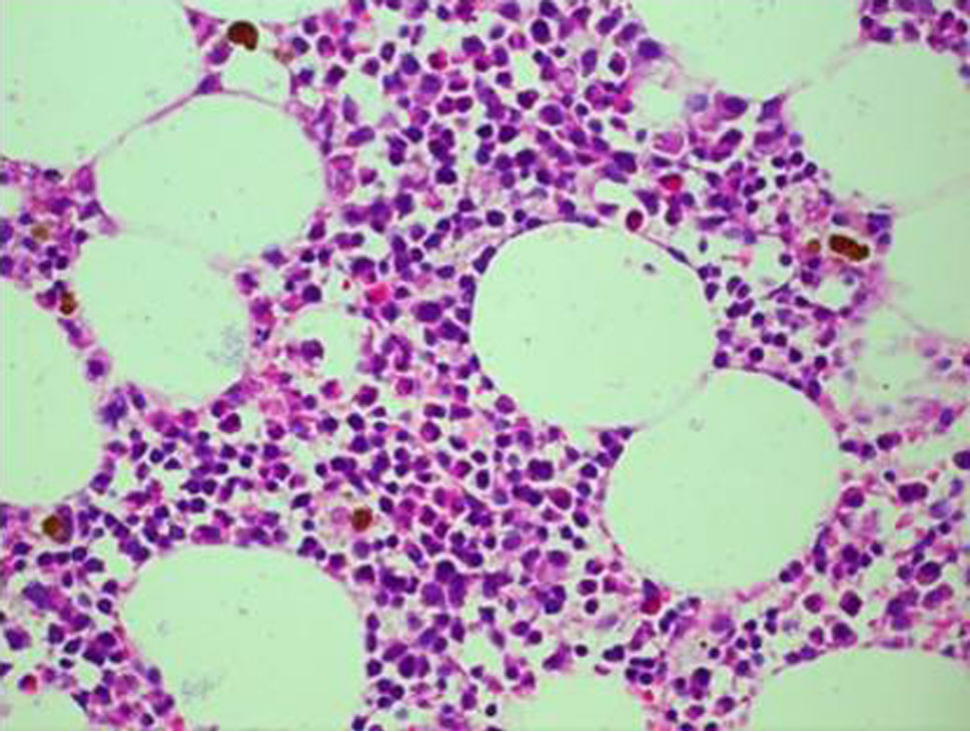
Vertebral biopsy showing gathered sheet-like atypical cells (HE staining; ×200)

**Fig. 9:**
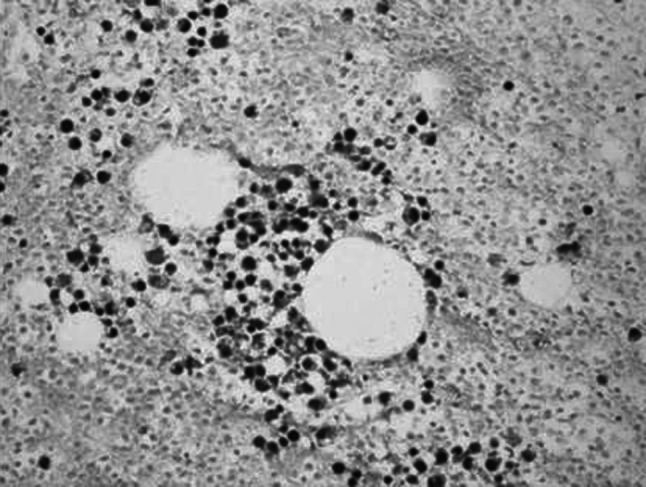
Vertebral biopsy showing gathered atypical cells and large surrounding areas of tissue necrosis (HE staining; ×200)

**Fig. 10:**
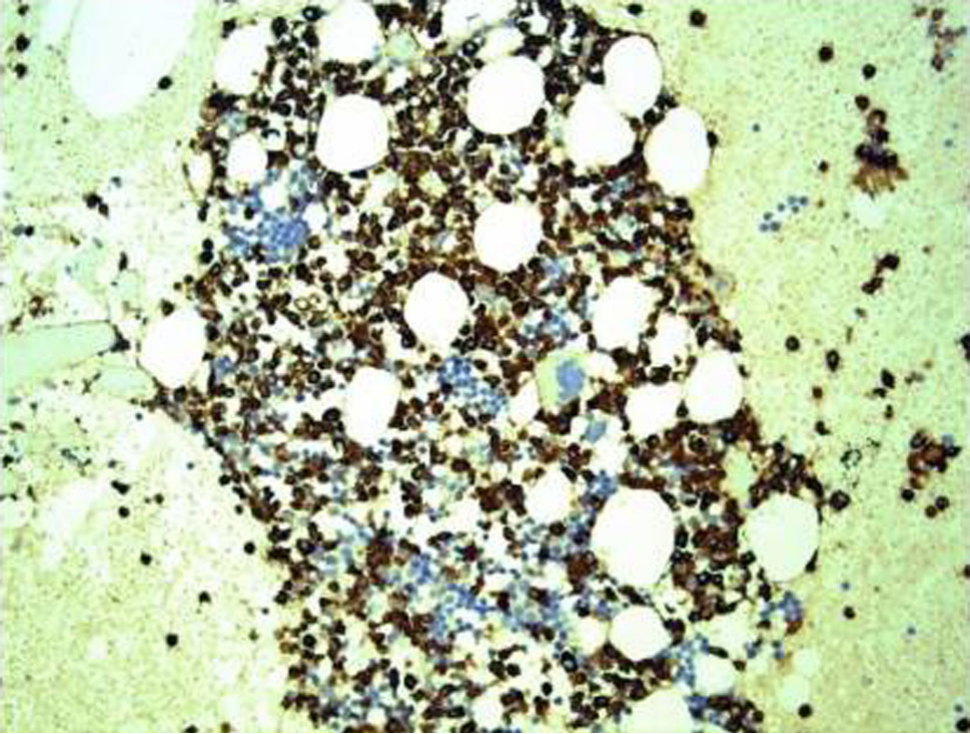
Myeloperoxidase (MPO) staining showing atypical cells (×200)

**Fig. 11:**
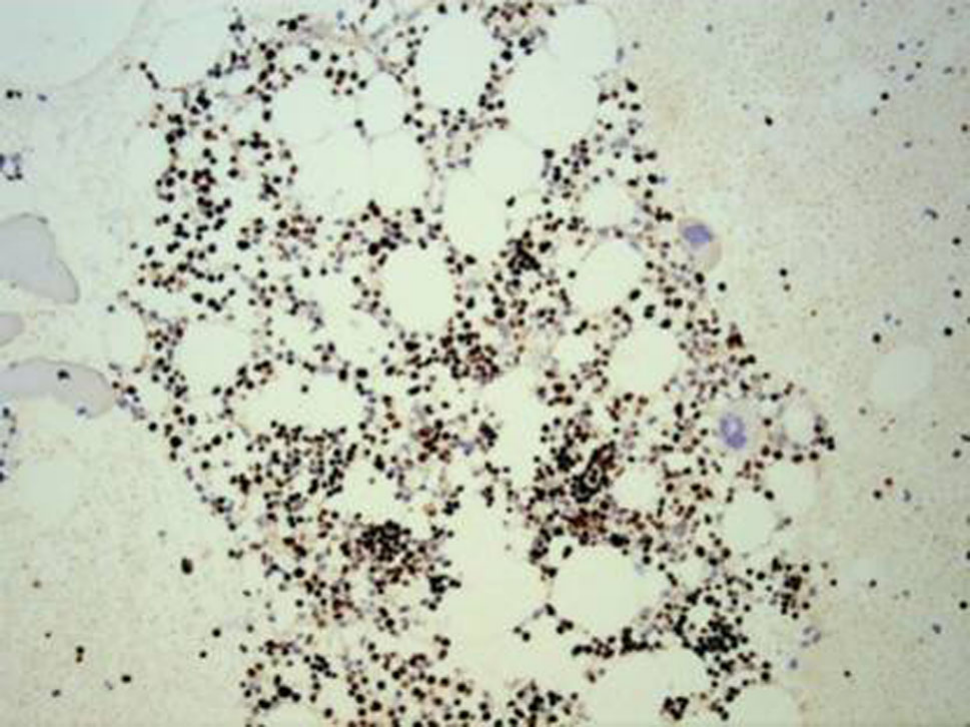
Ki-67 staining of atypical cell nucleus (×200)

No infection or severe bleeding occurred after the transplantation but hematopoietic recovery was slow, i.e., till +18d after the transplantation and the blood results were as follows: WBC: 1.28 × 10^9^/L; Hb: 61 g/L; PLT: 21 × 10^9^/L. Symptomatic and supportive treatments and intermittent G-CSF injections were given and the patient’s condition remained stable. At 3 months post transplantation, the patient complained of pain in lumbo-sacrum and the MRI prompted sheet-like abnormal signals of the 4th and the 5th lumbar vertebrae, and thus the leukemia infiltration was suspected (Fig. 4). The patient refused further examination and insisted on being discharged from the hospital and taking oral anodyne. At 4 months after the transplantation, the pain still persisted; the patient was unable to walk upright and stayed in bed. On re-admission, the patient was subjected to percutaneous puncture lumbar vertebral biopsy and lumbar vertebroplasty of 3rd, 4th, and 5th vertebrae; bone cement was injected into the damaged vertebra (Figs. 5, 6, 7). Bone biopsy showed sheet-like atypical cells, immunophenotyping showed CD20-, CD3 focally positive, Ki-67+ (40 %), MPO++, CD43+, PAX-5-, CD45RO+, CD38+, and thus extramedullary relapse of AML was considered (Figs. 8, 9, 10, 11). Afterwards, local radiotherapy was executed, i.e., radiotherapy of the vertebrae involved using linear accelerator for a total of 36 Gy.

At present, that is 10 months after the transplantation, the patient is found to be in a good general condition, however, with a mild waist pain and can walk. The routine blood examination showed: WBC: 3.72 × 10^9^/L; Hb: 101 g/L; PLT: 98 × 10^9^/L; myelography and FCM examinations showed a continuous complete remission of the marrow.

## Discussion

This patient in question has a history of breast cancer and had undergone chemotherapy. By marrow examination, the diagnosis of AML was clear, but FISH results revealed the deletion of chromosome 7, which associated with MDS. The marrow cytology showed hyperplasia whereas the typical AL always involves symptoms such as marked hyperplasia or hyperactivity plus the history of cytopenia before the diagnosis, so we suspected that the patient had AML transformed from MDS and which might be related to the breast cancer chemotherapy.

In regard to whether the highly intensive treatment can benefit older patients, different research studies have yielded discordant results. A large scale study reported that 70–79 years old patients that received highly intensive chemotherapy had not only shown a better tolerance but their CR and long-term survival rates were also significantly improved [5]. Allo-HSCT is a unique, potentially curative option as consolidation therapy regimen [6]. However, the traditional myeloablative conditioning regimen of forming donor’s complete chimerism has significant toxic side effects in older patients [7]. Notably, the reduced intensive conditioning regimen—a regimen with lower toxicity which is more suitable for older patients, brings the graft-versus-leukemia reaction by donor’s partial chimerism. But this patient did not have a suitable donor, however, the marrow showed complete remission just after one chemotherapy course with a good reactivity. Since a more intensive consolidation treatment was necessary, we tried through adopting auto-HSCT. After the surgery, the patient had slow hematopoietic recovery which related with the patient’s age and history of MDS [8, 9].

Although we closely monitored the patient’s myelography and excluded the relapse of hematopoietic system leukemia, the patient’s bone destruction indicated extramedullary relapse which was controlled after the treatment of local forming and radiotherapy. At 10 months after the transplantation, there was no medullary relapse or relapse manifestations of extramedullary leukemia. Furthermore, the hematopoietic recovery was delayed, as often observed for older patients with AML transformed from MDS. Following auto-HSCT, sustained marrow remission is rare, and also the extramedullary relapse in form of vertebral bone destruction is not common clinically. At the same time, clearing of leukemia clones may be difficult which is consistent with our analysis of the patient’s AML transformed from MDS as leukemia in older people has very low cure rate [10]. Despite all these setbacks, modalities such as local kyphoplasty and radiotherapy have benefited, and the patient remains to be in good general health status. Thus, the prime objective of prolonged survival was successfully achieved through the auto-HSCT in an elderly patient with AML transformed from MDS.
